# Experimental Studies on the Flammability and Fire Hazards of Photovoltaic Modules

**DOI:** 10.3390/ma8074210

**Published:** 2015-07-09

**Authors:** Hong-Yun Yang, Xiao-Dong Zhou, Li-Zhong Yang, Tao-Lin Zhang

**Affiliations:** 1 State Key Laboratory of Fire Science, University of Science and Technology of China, 96 Jinzhai Road, Hefei 230026, Anhui, China; E-Mails: yang007@mail.ustc.edu.cn (H.-Y.Y.); zxd@ustc.edu.cn (X.-D.Z.); ztlfire@mail.ustc.edu.cn (T.-L.Z.); 2 Collaborative Innovation Center for Urban Public Safety, 96 Jinzhai Road, Hefei 230026, Anhui, China

**Keywords:** photovoltaic fires, flammability, fire hazards, cone calorimeter

## Abstract

Many of the photovoltaic (PV) systems on buildings are of sufficiently high voltages, with potential to cause or promote fires. However, research about photovoltaic fires is insufficient. This paper focuses on the flammability and fire hazards of photovoltaic modules. Bench-scale experiments based on polycrystalline silicon PV modules have been conducted using a cone calorimeter. Several parameters including ignition time (*t*_ig_), mass loss, heat release rate (HRR), carbon monoxide (CO) and carbon dioxide (CO_2_) concentration, were investigated. The fire behaviours, fire hazards and toxicity of gases released by PV modules are assessed based on experimental results. The results show that PV modules under tests are inflammable with the critical heat flux of 26 kW/m^2^. This work will lead to better understanding on photovoltaic fires and how to help authorities determine the appropriate fire safety provisions for controlling photovoltaic fires.

## 1. Introduction

Solar energy is one of the most promising renewable energy resources. The United States advanced project of “one million solar roofs” in June 1997, which planned to install solar energy systems on one million roofs or other possible sites of buildings. Similar projects were also carried out in other countries such as Germany, Japan and China. Meanwhile, building integrated photovoltaic (BIPV) is in rapid development. The scale of BIPV in 2009 was $ 1.8 × 10^9^, and is expected to grow to $ 8.7 × 10^9^ in 2016 [1]. 

As an emerging technology installed on residential and commercial buildings, the security of BIPV is a prime concern. Therefore, it should be ensured that photovoltaic modules will not cause any damage to the buildings nor harm to the residents. However, fires in residential and commercial buildings are relatively common. Photovoltaic arrays mounted on buildings might worsen the pre-existing level of fire hazards. This is because photovoltaic (PV) modules could modify the propagation of fire outside or through the building. It might interfere with the smoke and venting system, which will hamper the fire extinction operations as well as induce a further hazard through electrical shock for firefighters. Moreover, many of the PV systems on buildings are of sufficiently high voltages (300 to 1000 Volts DC) [2,3], which means that they may start a fire themselves.

PV modules are closely related to lives and properties. Consequently, there have been lots of efforts to establish rigorous safety standards to mitigate the potential risks. PV modules are tested to either IEC 61730-2 [4] or UL 1703 [5] or both. These two standards have similar requirements, including fire-resistant, hot spot, and temperature tests. They both have effectively restricted designs that minimize the spread of fires. As a special roof deck, PV modules may also be tested to UL 1256 [6] which requires a direct fire heating at 760 °C, for 30 min. Italian National Fire Services Guidelines provide a procedure to assess and alleviate fire risks caused by PV arrays located on buildings [7]. Article 690.11 of the 2014 National Electrical Code [8] requires detection of series arc faults in either or both of the dc source circuits, or the dc output circuits [3]. These standards or guidelines are still being improved by many researchers. For example, the causes of PV fires have been investigated by Wohlgemuth *et al.* [2]. They found that hot spots, high series resistance and arcing are three typical ways that a module can be overheated to start a sustainable fire within the module. Thus, they modified IEC 61730-2 [4] to improve the way of testing a module’s potential to cause a fire. 

Even with all these efforts, severe building fires involving PV arrays have been reported in the past few years, such as the fire in LaFarge (WI, America) in May 2013, of which the big fire began with a small fire from the rooftop PV system. As a result, it is still worthwhile to study the flammability and fire hazards of PV modules in depth, which is the motivation of the current study.

## 2. Analysis of the Flammability of PV Modules

When PV modules are on fire, foam and powder are not preferred options. As PV arrays are often sloped, foam or powder could simply slide off. Moreover, many PV systems are of high voltages (300 to 1000 Volts DC), which are rather life-threatening. During a fire event, it is not possible to turn off the whole photovoltaic power system in order to guarantee that all the components are de-energized. In fact, these systems are alive as long as there is light. Thus water jets are also of limited usage in such situations because of their conductibility. Fortunately, there is research demonstrating that water jets can still be used in PV fires by respecting a specific safety distance [9]. However, the safety distance strongly depends on the type of nozzle, on the water pressure and on the water flow rate, which should be determined based on specific conditions. International guidelines for firefighters to cope with the photovoltaic fires are not well established for now. Thus, more attention should be paid to relative research. The encapsulant of PV modules, *i.e.*, ethylene-vinyl acetate copolymer (EVA), is the main combustible component. There have already been lots of studies about combustion characteristics and flame retardancy of EVA. Bonnet *et al.* [10] developed a new EVA-based hybrid material containing silicon and phosphorus to improve fire retardancy of EVA. Ohuchi *et al.* [11] conducted experiments using a cone calorimeter and found that the fluoroplastic has more advantages as a fire-proofing cell encapsulation material than EVA. 

When it comes to fire hazard assessment by taking a PV module as a whole, only a little research has been done. Fthenakis *et al.* [12] have conducted experimental investigations on both emissions and redistribution of elements in CdTe PV modules during fires. They found that the actual Cd loss during fires would be insignificant. Other research mainly focuses on fire resistance testing and fire preventing of PV modules [2,13,14]. On the other hand, heat intensity properties and toxic gases (CO, CO_2_) emitted by PV modules have not been investigated yet. The current work tries to assess the hazards of heat released by combustion of PV modules and toxicity of gases emitted through bench-scale experiments.

As reported in the literature [15,16], tests with a cone calorimeter would be useful in understanding the fire behaviour of materials. The results of both heat and smoke aspects can be applied to fire assessment with fire models to complement full-scale burning tests. In this paper, photovoltaic fire behaviors are evaluated with a cone calorimeter. Fire behaviour, thermal hazards and toxicity of gases released by PV modules are investigated.

## 3. Experimental Section

### 3.1. Materials

**Figure 1:**
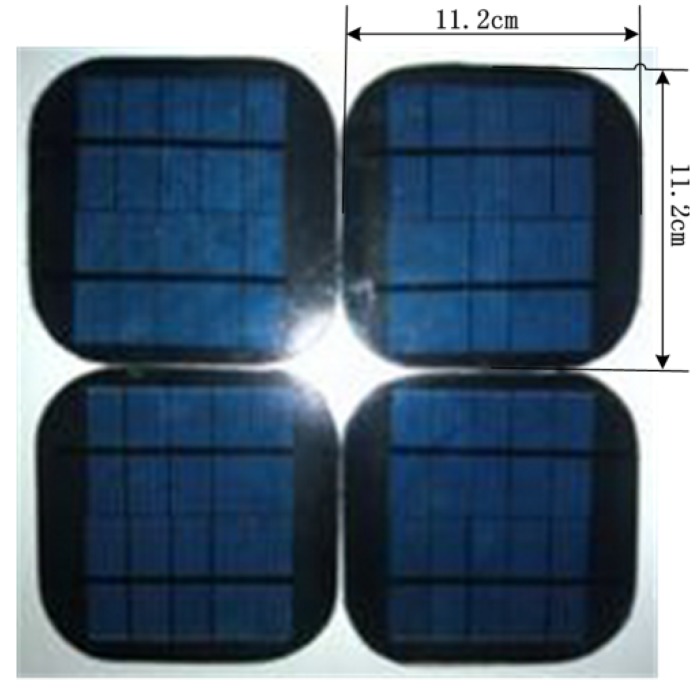
PV modules used in the tests.

**Figure 2:**
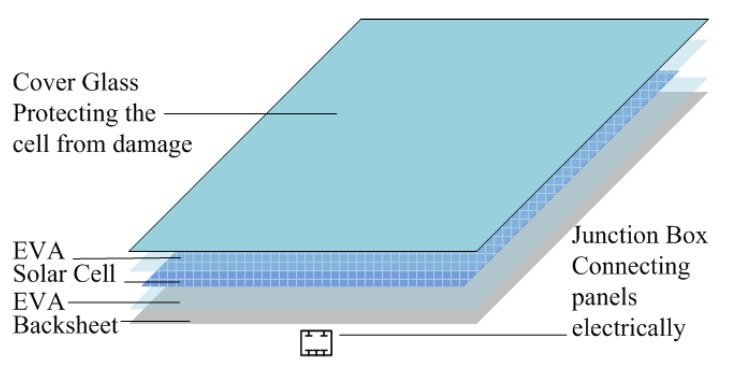
Schematic view of a PV module.

This study is based on small typical polycrystalline silicon PV modules, 11.2 cm × 11.2 cm × 0.39 cm in size, which are shown in Figure 1. The specimens, fabricated in 2014 and bought on the market, have been certificated by IEC 61730-2 [4] and UL 1703 [5]. The initial mass of a sample is equal to 135 ± 2 g. The PV module has a five-layer structure, as shown in Figure 2. A layer of low-iron glass is typically used as the top layer of a PV module, which provides mechanical strength. It protects the PV module from physical damage and allows light to transmit into the solar cells. EVA film is used to encapsulate the PV module, which is also the main combustible component. The third-layer solar cell converts sunlight to electricity. And the back-sheet protects the PV module from ultraviolet and moisture, which is also flammable. In this investigation, the PV module is considered as a whole unit. The test specimens were placed in a constant temperature and humidity incubator at 65% relative humidity (a typical ambient relative humidity) and 23 °C (a common air temperature) and weighed at regular intervals until equilibrium was reached (about 24 h in this experiment). This job ensures that all samples have the same initial conditions.

### 3.2. Test Method

**Figure 3:**
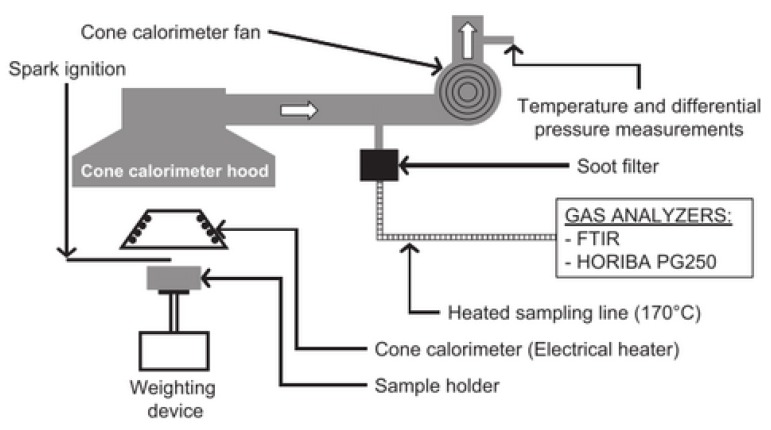
Experimental device schematic layout [16].

The cone calorimeter is one of the most effective bench-scale methods for studying flammability properties of materials. Its results have been found to correlate well with those obtained from large-scale fire tests and can be used to predict the behaviour of materials in real fires [17]. A cone calorimeter, as shown in Figure 3, is used in tests to provide external radiation. The details about standard test procedure for the cone calorimeter are discussed in [18].

Bench-scale experiments show that flame heat flux is in the range of 20–70 kW/m^2^ [19,20,21]. Babrauskas *et al.* [22] discussed the heat flux for bench-scale tests. He found that the heat flux of 25–50 kW/m^2^ is proper for most research purposes. In the preliminary experiments of this work, 45 kW/m^2^ was the biggest controllable radiative heat flux and 25 kW/m^2^ was not high enough to ignite samples. Therefore, 28, 30, 35, 40 and 45 kW/m^2^ are chosen in the experiments. That is, there are five tests on the same kind of specimens with five different heat fluxes. Tests were repeated at least two times for each condition to ensure reproducibility. The uncertainty on heat flux is about 5% [23]. The end of test time for calculation purposes is based on the mass loss rate criterion, *i.e.*, the time that the average mass loss rate drops to lower than 1 g/m^2^s in one-minute period. During tests, the following parameters are measured: ignition time (*t*_ig_), mass loss, heat release rate (HRR), carbon monoxide (CO) and carbon dioxide (CO_2_) concentration. Some parameters of PV modules are calculated: critical heat flux, mass loss rate, total heat release rate and fractional effective dose (FED). Fire behaviour, fire hazards, and the toxicity of gases released by PV modules are assessed based on these parameters.

## 4. Results and Analysis

### 4.1. Experimental Phenomena

**Figure 4:**
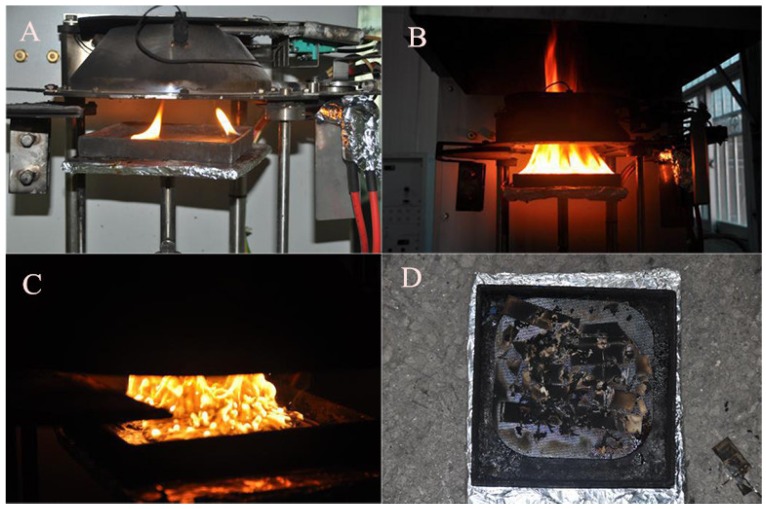
Combustion process of PV modules. (**A**) incipient stage; (**B**) development stage (**C**) fully burning stage (**D**) decline stage.

Some interesting phenomena have been observed during tests. Exposed to the conical radiation source for a period of time, the back-sheet of the PV module is heated in order to melt and vapors rise around the PV module. Then, vapors are ignited and flames appear as shown in Figure 4A. That is, the PV fire is a kind of multiple fire burning in the beginning stage. As burning accelerates, flames merge into a single one as shown in Figure 4B. It is important to note that a cluster of bubbles appears in the combustion process showing in Figure 4C, illustrating the combustion is rather intense. Cover glass in the PV module breaks into pieces after the burning as shown in Figure 4D, which makes PV fires harder to deal with, having a great possibility to injure firefighters. Moreover, when EVA melts under external heat fluxes, there will be dripping behavior during real photovoltaic fires, which will facilitate fire propagation. 

### 4.2. Fire Behaviour

Ignition time (*t*_ig_) is one of the critical parameters for reaction of materials to fire. The higher the ignition time, the longer it takes to heat up and ignite a fire. The ignition time (*t*_ig_) of a PV module is experimentally determined as the delay from the test start to the appearance of a sustainable flame on the sample surface.

**Figure 5:**
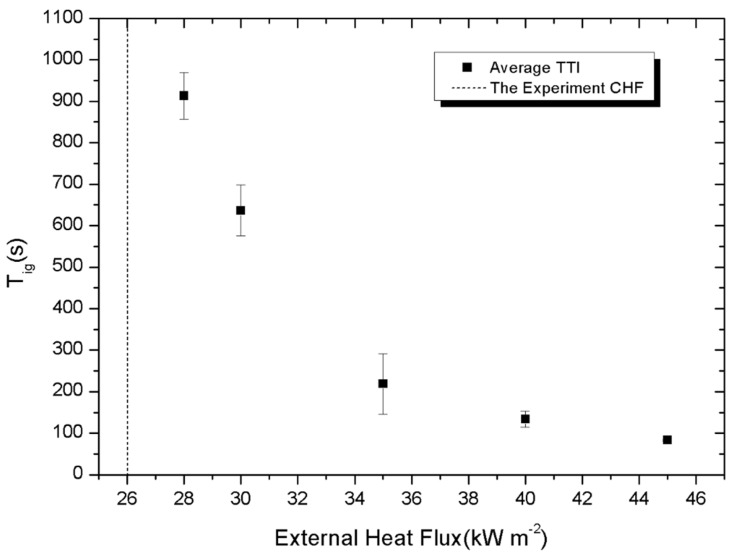
Evolution of ignition time *versus* heat flux.

Figure 5 presents the evolution of ignition time (*t*_ig_) as a function of heat flux. It can be seen in Figure 5 that the increase in heat flux induces a rapid decrease in ignition time. For example, the ignition time of the samples decreases from 913 to 83 s when the heat flux increases from 28 to 45 kW/m^2^. The ignition time curve approaches the vertical asymptote which intercepts the x-axis at 26 kW/m^2^, corresponding to the critical heat flux (CHF). CHF is defined as the minimum value of heat flux below which no flame occurs. In addition, CHF is another parameter to assess fire behaviour. In residential fires, the typical flame temperature for roof fires is in the range of 800–900 °C. While in fires involving the whole house, flame temperature is in the range of 900–1000 °C as measured in [24]. With CHF of 26 kW/m^2^, which can be easily reached by flame heat flux, the PV modules used in this test are flammable. Theoretically, the ignition time is calculated by Equation (1) [25] or Equation (2) [23] for thermally thin materials and thermally thick materials respectively:

(1) $$t_{ig}=\frac{\rho C_{p}d(T_{ig}-T_{\infty })}{{\dot{q}}^{\prime \prime }}$$

(2) $$t_{ig}=\frac{\pi }{4}(\lambda \rho C_{p}){\left(\frac{T_{ig}-T_{\infty }}{{\dot{q}}^{\prime \prime }}\right)}^{2}$$

**Figure 6:**
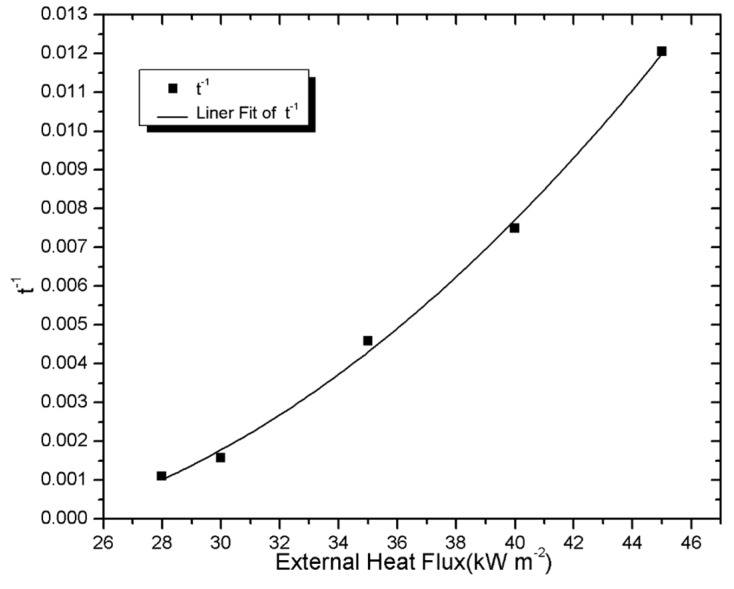
Ignition times plotted assuming thermally thin conditions.

**Figure 7:**
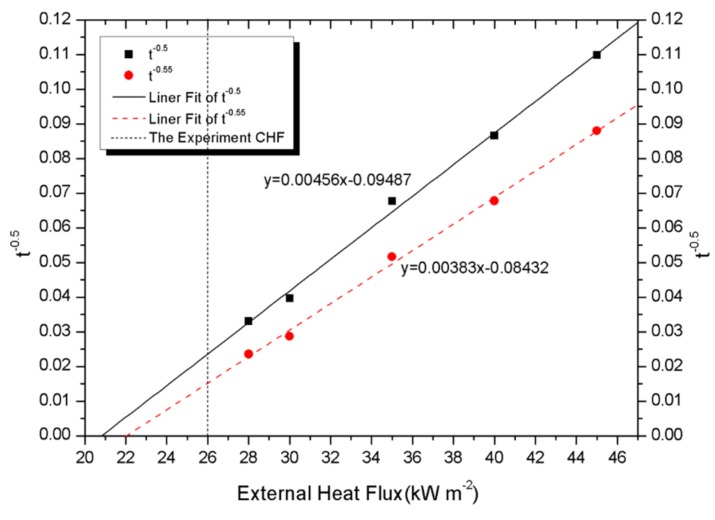
Ignition times plotted assuming thermally thick conditions.

Figure 6 and Figure 7 (square) display $t_{\text{ig}}^{-1}$ and $t_{\text{ig}}^{-0.5}$ *versus* external heat flux, respectively. The curve of $t_{\text{ig}}^{-1}$ as function of the external heat flux is not linear as shown in Figure 6. Conversely, $t_{\text{ig}}^{-0.5}$ plots (square in Figure 7) *versus* external heat flux can be fitted by a straight line with the fitting degree of 0.995. These observations allow us to conclude that the PV module is thermally thick. Indeed, while a thermally thick solid material is heated up, a temperature gradient is observed inside the sample. On the other hand, the temperature inside the sample is uniform in a thermally thin material.

**Figure 8:**
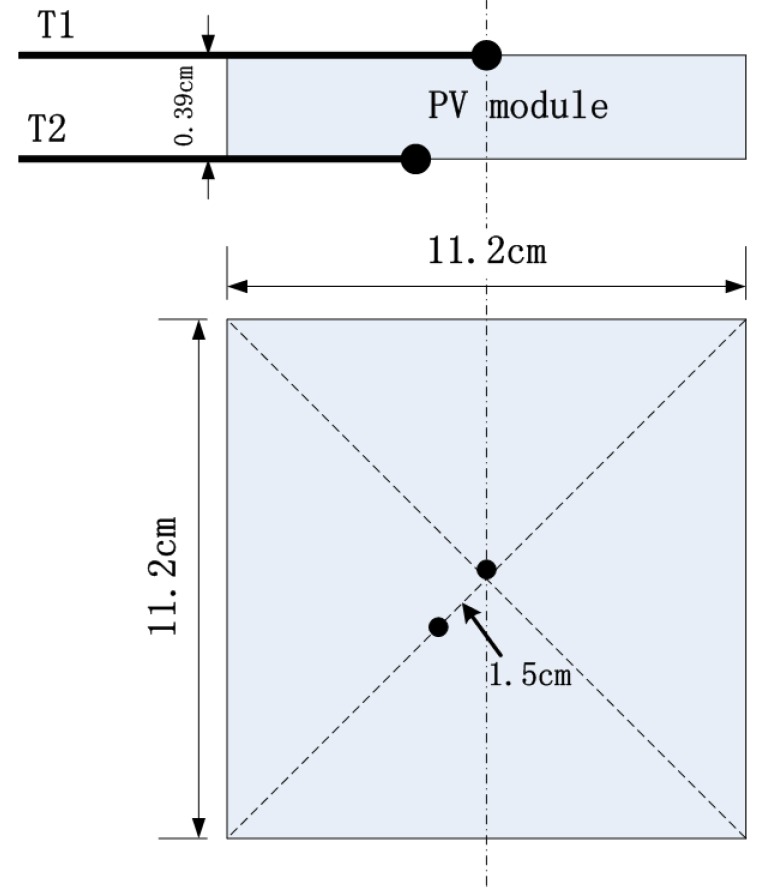
Thermocouples setup.

In order to verify the thermally thick property of PV modules, some tests were conducted to find if there are temperature gradients in them. Two thermocouples (type K, 1 mm in diameter) were mounted to measure the temperature profiles. One of thermocouples (T1) is located at the centre of the surface exposed to the heat flux, and the other one (T2) is 1.5 cm apart from the centre of the bottom of the sample as shown in Figure 8. Experiments were conducted under 25 kW/m^2^ to eliminate the interference of flames.

**Figure 9:**
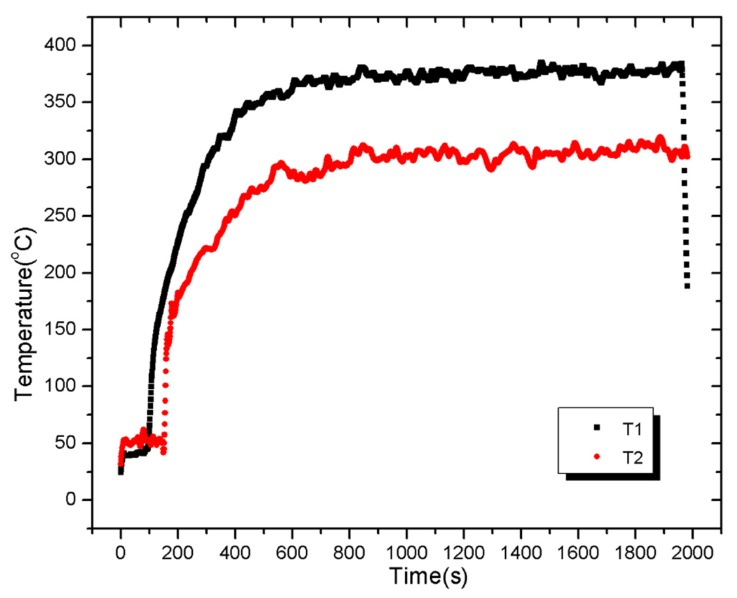
Temperature profiles of T1 and T2 under external heat flux of 25 kW/m^2^.

Figure 9 shows a typical profile of T1 and T2 under external heat flux of 25 kW/m^2^. A maximum difference of about 100 °C inside a given sample is observed in Figure 9. This gradient of temperature inside the material confirms the thermally thick property of the PV module. The temperature measurements were repeated twice, within the maximum deviation of ±20 °C.

Considering surface radiation thermal losses ($\sigma \varepsilon (T_{s}^{4}-T_{\infty }^{4})$) and convection thermal losses ($h(T_{s}-T_{\infty })$), Janssens [26] modified the relation between ignition time and external heat flux as $t_{\text{ig}}^{-0.55}\propto {\dot{q}}^{\prime \prime }$ for thermally thick materials, which is also shown in Figure 7 (circle). The fitted lines of $t_{\text{ig}}^{-0.5}$ and $t_{\text{ig}}^{-0.55}$ reach intersection points with the x-axis of approximately 20.8 kW/m^2^ and 22.02 kW/m^2^ respectively, which represent the theoretical CHF of PV modules. By contrasting with the experimental critical heat flux of 26 kW/m^2^ represented by a vertical dashed line in Figure 7, a conclusion can be made that the modification of Janssens is reasonable. It makes the theoretical calculation closer to the experimental one. The difference between the experimental and theoretical CHF can be due to the non-linearity of the ignition time for external heat flux near the experimental CHF (where the solid material is thermally thin compared to the time of thermal transfer).

### 4.3. Fire Hazards

The heat release rate (HRR) is the most significant parameter for materials’ fire hazards evaluations. The HRR represents the rate of thermal energy generated by combustion, which controls the growth rate of fire as well as the amount of smoke and gaseous effluents generated. Heat released from a single burning item might be strong enough to ignite adjacent items, causing fire propagation. Moreover, the peak heat release rate (pkHRR) is the parameter that best expresses the maximum intensity of a HRR curve. This parameter (with a relative uncertainty of ±5%) is calculated by using the oxygen consumption calorimeter technique based on the ISO5660-1 standard [27] with equations described in detail in [18].

Petrella [28] mentioned that a combination of parameter *x* (an indication of propensity to flashover) and total heat release (THR) would give reasonable indications of fire thermal hazards of the material. The rating system presented by Petrella is shown in Table 1. The shortcoming of Petrella’s method is that two separate classifications are presented by using two parameters (*x* parameter and THR, separately). That is, consistent fire hazards rank might not be gained with this method.

Chow [15,29] used Petrella’s method for the assessment of fire hazards of video compact disc (VCD) materials and sandwich panels. Bakhtiyari *et al.* [17,30] used the same method to investigate fire hazards of expanded polystyrene and polyurethane foams with cone calorimeter. All those studies show that Petrella’s method for fire hazard assessment is sensible and effective. Moreover, these research achievements can complement the incapability of this method in calculating consistent fire hazards rank with more references.

**Figure 10:**
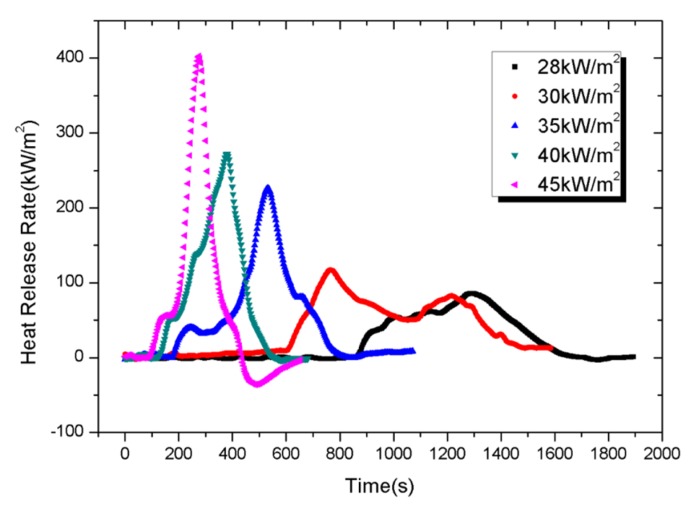
Heat release rate (HRR) of PV modules as a function of time.

Figure 10 shows the transient evolutions of the HRR at five irradiance levels (28, 30, 35, 40 and 45 kW/m^2^). Transient evolution of HRR depends strongly on the irradiation level. Globally, pkHRR increases from 85 to 402 kW/m^2^ with external heat flux increasing from 28 to 45 kW/m^2^.

**Table 1:** Evaluation system proposed by Petrella.

Values	*x* parameter	Total Heat Release (THR)
0.1–1.0	Low risk to flashover	Very low risk to heat contribution
1.0–10	Intermediate risk to flashover	Low risk to heat contribution
10–100	High risk to flashover	Intermediate low risk to heat contribution
100–1000	-	High risk to heat contribution

As discussed by Petrella [28], parameter *x* is introduced as an indication of propensity to flashover, which can be calculated by the ratio of the peak of heat release rate (pkHRR) to time to ignition (TTI):

(3) $$x=\frac{\text{pkHRR}}{\text{TTI}}$$

THR (in MJ/m^2^) is calculated by integrating the curve of the HRR over time as shown in Equation (4):

(4) $$\text{THR}=\int_{0}^{\infty }\left(\text{HRR}\right)\text{dt}$$

*x* parameter and THR of PV modules are shown in Table 2. With the *x* parameter in the range of 0.09–4.84, PV modules have a low risk to flashover under external heat fluxes lower than 30 kW/m^2^ and an intermediate risk to flashover under external heat fluxes between 35–45 kW/m^2^. In addition, it is obvious that PV modules used in tests will give intermediate risk with the THR in the range of 38–57 MJ/m^2^. Actually, the thicknesses of large-scale PV modules used in building roof are about 5–10 times thicker than the experimental small ones, whose THR can be high enough to reach high risk.

**Table 2:** Test results and thermal hazard classification.

Heat flux (kW/m^2^)	Derived data			
	TTI (s)	pk HRR (kW/m^2^)	x parameter (kW/m^2^s)	THR (MJ/m^2^)
28	913	85	0.093	38.270 (Intermediate risk)
30	636	116	0.182 (low risk)	56.736 (Intermediate risk)
35	218	226	1.037 (Intermediate risk)	50.069 (Intermediate risk)
40	133	272	2.045 (Intermediate risk)	48.524 (Intermediate risk)
45	83	402	4.843 (Intermediate risk)	45.481 (Intermediate risk)

### 4.4. Toxicity of Gases

**Figure 11:**
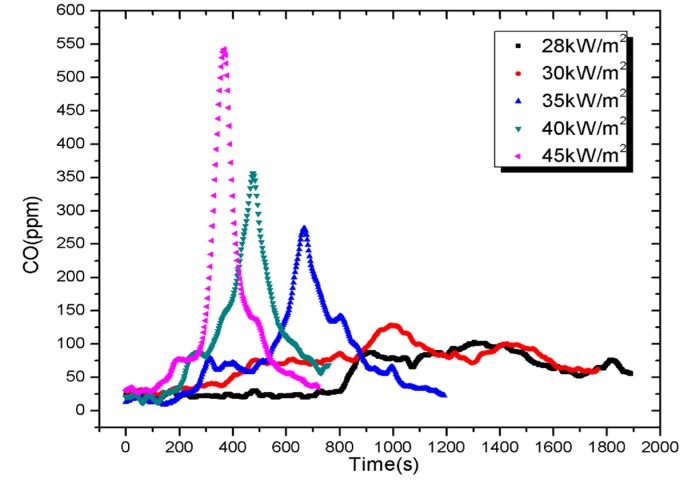
Carbon monoxide concentration as a function of experimental time.

**Figure 12:**
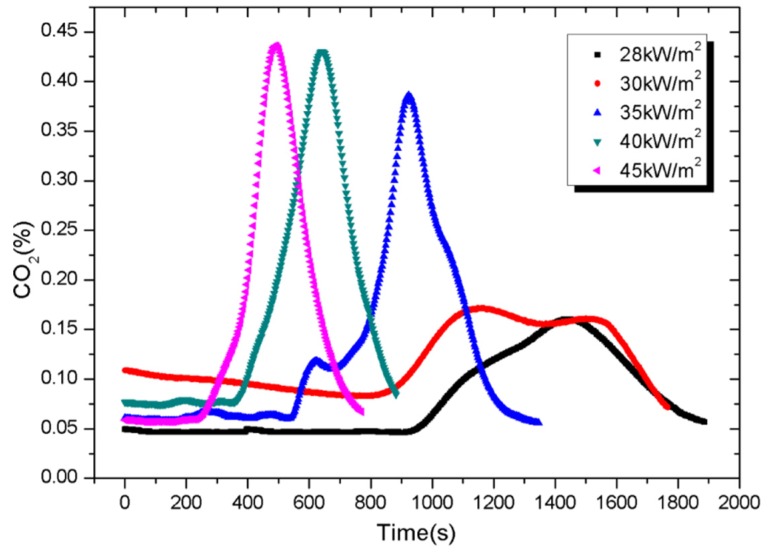
Carbon dioxide concentration as a function of experimental time.

Results of CO and CO_2_ concentration, detected by the cone calorimeter [31], are shown in Figure 11 and Figure 12. The standard uncertainties of both gases are ±8%. Taking the test under external heat flux of 45 kW/m^2^ for instance, CO emitted from samples increases significantly after the ignition time of 83 s, because the CO and smoke increase as flames appear on materials. It is observed that the maximum concentration of CO increased from 101 to 522 ppm when the heat flux increases from 28 to 45 kW/m^2^. Meanwhile, the maximum concentration of CO_2_ varies from 0.159% to 0.436%. Both parameters have same curve shapes with HRR for a given external heat flux.

Since only CO and CO_2_ are measured, the peak fractional effective dose (FED) is calculated from the peak concentration of CO and CO_2_ denoted by [CO], [CO_2_] and their toxic potency *LC*_CO_, *LC*_CO2_ as shown in Equation (5):

(5) $$\text{FED}=\frac{\left[\text{CO}\right]}{{LC}_{\text{CO}}}+\frac{\left[\text{CO}\right]}{{LC}_{{\text{CO}}_{2}}}$$

In the above equation, the LC_CO_ value is the reference concentration of CO, which causes death when inhaled for a specific amount of time, typically 30 min. The same is true for ${LC}_{{\text{CO}}_{2}}$. FED is a parameter for toxic gas evaluation. The higher the value of FED, the stronger the toxicity becomes. Toxic gases can cause death when FED value is about 1. Since ${LC}_{{\text{CO}}_{2}}$ is very large, FED is calculated only from [CO] by taking *LC*_CO_ as 5000 ppm [15] as shown in Equation (6):

(6) $$\text{FED}=\frac{\left[\text{CO}\right]}{5000}$$

As shown in Table 3, values of FED for tests under five external heat fluxes are low, within the range of 0.02–0.108. It means that CO emitted by PV modules is negligible.

**Table 3:** Test results of gases.

Heat flux (kW/m^2^)	Peak CO (ppm)	FED = [CO]/5000
28	101	0.0202
30	128	0.0256
35	274	0.0548
40	356	0.0712
45	542	0.108

### 4.5. Mass Loss and Mass Loss Rate

**Figure 13:**
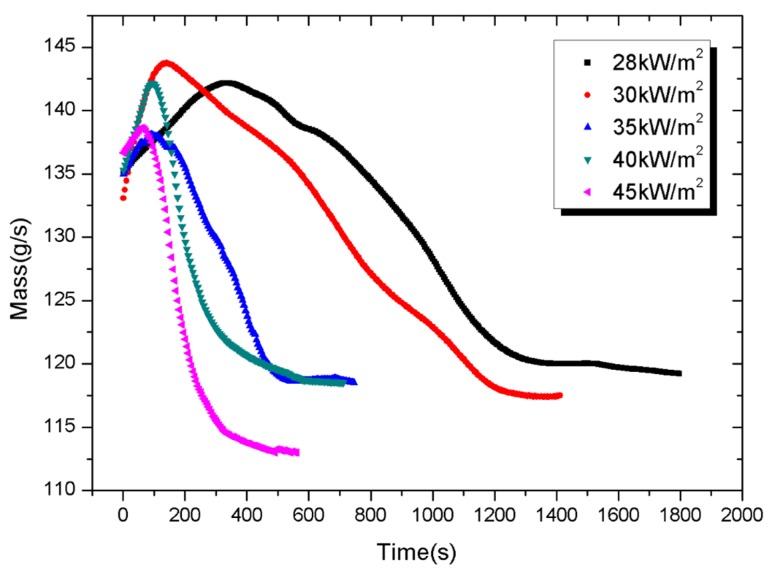
Mass *versus* experimental time.

**Figure 14:**
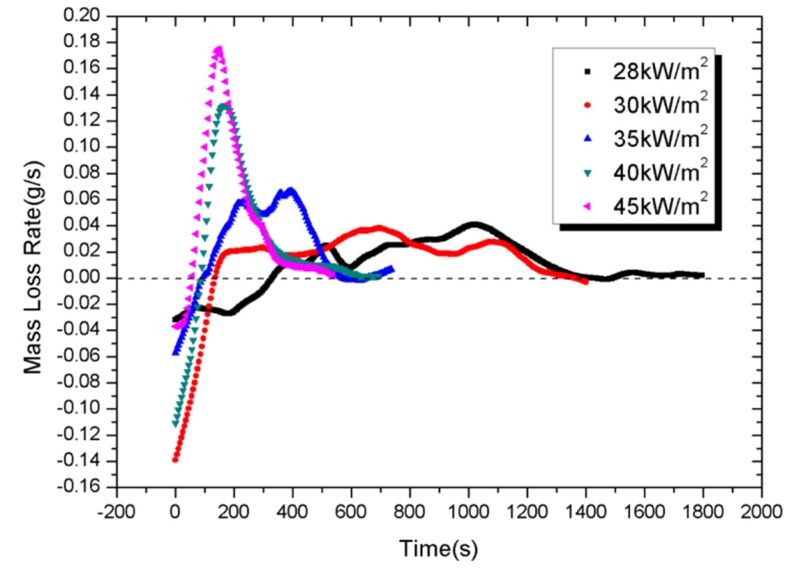
Mass loss rate *versus* experimental time.

The mass and mass loss rate (MLR) evolution as functions of time under different external heat fluxes are represented in Figure 13 and Figure 14, respectively, during the cone calorimeter experiments. The standard uncertainties of the measured mass loss are ±10%. As shown in Figure 13, samples have shown an obvious weight increase during the inception phase, possibly due to oxidation. In addition, the PV module under lower heat flux experienced a longer weight-increasing period. That is because oxidized products which are relatively heavier begin to decompose when temperature grows to a certain level. The remaining of the PV module under higher heat flux is less, indicating a more complete burning.

MLR is calculated from Equation (7) by the difference approach. The widths and the intensities of the MLR curve depend strongly on external heat flux. In fact, an irradiation level increase moves curve shapes towards shorter experimental time (e.g., around 1800 s at 28 kW/m^2^ against 600 s at 45 kW/m^2^); also increases the maximal intensity of the MLR peak (e.g., around 0.18 g/s at 45 kW/m^2^ compared to 0.04 g/s at 28 kW/m^2^). These observations show that the increasing heat flux decreases the resistance of PV modules and accelerates their combustion.

(7) $$\text{MLR}=\frac{m_{t}-m_{t-\Delta t}}{\Delta t}$$

## 5. Conclusions

Aiming to get a better understanding of PV fire behavior and hazards, five tests on the same kind of specimen with different heat fluxes have been conducted using a cone calorimeter. Several parameters of PV modules such as ignition time, critical heat flux, mass loss rate, heat release rate and toxicity of gases were systematically measured and calculated.

It is observed that exposing the materials to high heat fluxes would be very dangerous. The PV modules under tests could be ignited by heat fluxes greater than 26 kW/m^2^, which can be easily reached by flame heat fluxes in real fires. Furthermore, a temperature gradient is observed inside the sample, showing the thermally thick property of PV modules. CO and CO_2_ emitted by PV modules are negligible. With the THR in the range of 38–57 MJ/m^2^, PV modules used in tests are at intermediate risk. However, in residential fires, the typical flame temperature is in the range of 800–900 °C for roof fires and in the range of 900–1000 °C for fires involving the whole house. Furthermore, the thickness of big-size PV modules used in building roof is about 5–10 times thicker than small experimental ones. That is, PV modules mounted on buildings can be much more dangerous when exposed to real fires than these experimental ones.

Special care must be taken in designing fire safety provisions for buildings with photovoltaic systems. Parameters deduced from the cone calorimeter might be useful for setting up regulations to assess photovoltaic fires. Moreover, full-scale burning tests and tests for photovoltaic fire propagation will be carried out in the future studies to give more references.

## Nomenclature

*t*_ig_: ignition delay, s
ρ: density, kg/m^3^
C_p_: thermal capacity, kJ/(g K)
d: thickness of samples, m
T_∞_: ambient temperature, K
${\dot{q}}^{\prime \prime }$: external heat flux, kW/m^2^
λ: thermal conductivity, kW/(mK)
[CO]: carbon monoxide concentration, ppm
[CO_2_]: carbon dioxide concentration, ppm
*T*_ig_: ignition temperature, K
